# Preparedness for the Digital Transition in Healthcare: Insights from an Italian Sample of Professionals

**DOI:** 10.3390/healthcare13202556

**Published:** 2025-10-10

**Authors:** Valentina Elisabetta Di Mattei, Gaia Perego, Francesca Milano, Federica Cugnata, Chiara Brombin, Antonio Catarinella, Francesca Gatti, Lavinia Bellamore Dettori, Jennifer Tuzii, Elena Bottinelli

**Affiliations:** 1 School of Psychology, Vita-Salute San Raffaele University, 20132 Milan, Italy; 2 Clinical and Health Psychology Unit, IRCCS San Raffaele Scientific Institute, 20132 Milan, Italy; 3 Department of Psychology, University of Milan-Bicocca, 20126 Milan, Italy; 4 University Centre for Statistics in the Biomedical Sciences (CUSSB), Vita-Salute San Raffaele University, 20132 Milan, Italy; 5 Villa Erbosa S.p.A., Gruppo San Donato, 40129 Bologna, Italy

**Keywords:** digital transition, impostor syndrome, burnout, healthcare workers

## Abstract

**Background:** The digital transition is reshaping healthcare systems through the adoption of telemedicine and electronic health records (EHRs). While these innovations enhance efficiency and access, their implementation unfolds within overstretched organizational settings characterized by workforce shortages, bureaucratic demands, and heightened psychosocial risks. Burnout, impostor syndrome, and the quality of organizational support have thus become pivotal constructs in understanding healthcare professionals’ digital preparedness. **Methods**: A cross-sectional online survey was conducted among 111 professionals employed at two San Donato Group facilities in Bologna, Italy. The battery included socio-demographic and occupational data, perceptions of digitalization, and validated instruments: the Maslach Burnout Inventory (MBI), the Clance Impostor Phenomenon Scale (CIPS), and the Work Organization Assessment Questionnaire (WOAQ). Descriptive analyses were complemented by Classification and Regression Trees (CART) to identify predictors of perceived digital preparedness. **Results:** Most respondents (88%) acknowledged the relevance of digitalization, yet 18% felt unprepared, especially women and administrative staff. Burnout levels were high, with 51% reporting emotional exhaustion, most notably among nurses and female participants. Impostor syndrome affected 43% of the sample, with nurses exhibiting the highest prevalence. CART analysis identified emotional exhaustion, impostor syndrome, and age as principal discriminators of digital preparedness. **Conclusions**: Our findings highlight the role of emotional exhaustion, impostor syndrome, and age in shaping perceived digital preparedness, underscoring the need for tailored training and supportive practices to ensure a sustainable digital transition.

## 1. Introduction

The digital transition in the healthcare sector represents one of the most significant innovations in recent years, profoundly transforming the operational methods and responsibilities of healthcare professionals [1]. This transformation was markedly accelerated by the COVID-19 pandemic, which forced a rapid adoption of digital tools to ensure care continuity amid unprecedented constraints [2]. The digital transition refers to the adoption of digital technologies to benefit society, offering substantial opportunities in the healthcare domain, provided that adequate infrastructure and proper training are in place [3]. By leveraging digital tools, this transition enables the overcoming of traditional boundaries (e.g., geographical distance or the inability to attend in-person visits due to health conditions) while fostering the development of new competencies to improve organizational efficiency [4]. Digital innovation has introduced new models of medical practice to address care delivery challenges and improve clinical outcomes [1]. Specifically, these changes are linked to the use of the internet and digital technologies and their relationship with new therapies and best practices to optimize healthcare procedures [3]. Among the most impactful tools, telemedicine and electronic health records (EHRs) have transformed care delivery and documentation, enabling remote access to services, streamlining communication, and standardizing data management [3,5,6,7].

However, adopting these technologies has led to significant changes in clinical workflows, demanding new technical skills from healthcare professionals. This transformation unfolds within an already pressured work environment, marked by staff shortages, growing patient demands, complex health conditions, and administrative burdens. Such stressors have a significant impact on the healthcare workers’ mental well-being [8]. The management of health information, the need to ensure data security, and the implementation of new practices all require increasingly specialized competencies, which heighten the sense of responsibility and contribute to occupational stress [3,6].

Within this context, several psychosocial and organizational factors emerge as particularly relevant because they influence healthcare professionals’ capacity to engage with digital transition. Burnout and stress undermine both professionals’ well-being and their ability to adapt to change [8,9,10,11,12,13]. Within these dynamics, additional psychosocial and organizational constructs become highly relevant. Impostor syndrome, defined as persistent self-doubt and the fear of being exposed as inadequate [14], has been consistently linked to depression, anxiety, burnout, and reduced professional confidence [15,16,17], and may hinder openness to new technologies. Likewise, bureaucratic workload represents a critical concern in healthcare, as the time and energy absorbed by administrative demands often come at the expense of direct patient care, innovation, and training opportunities [18]. Beyond these vulnerabilities, organizational dimensions such as the quality of relationships with colleagues and management, perceived recognition and support, workload balance, and the adequacy of the work environment warrant attention, given their impact on psychological well-being [19]. Indeed, prior research has shown that workload, job burnout, and turnover intention are negatively associated with healthcare quality, while job satisfaction moderates these relationships [20] and, consequently, on professionals’ ability to engage with innovation. In line with this, supportive organizational environments—characterized by recognition, collegial relationships, and administrative support—can mitigate stress and promote engagement with innovation [21,22]. These factors provide a broader picture of how organizational conditions can shape professionals’ preparedness for digital transition.

Against this backdrop, the present study investigates the interplay between digital transition, psychological well-being, and organizational conditions among Italian healthcare professionals. Specifically, we examine how socio-demographic characteristics (gender, age, and professional role), psychological variables (impostor syndrome, emotional exhaustion, and other burnout dimensions), and perceptions of organizational support shape healthcare professionals’ perceived preparedness for the digital transition. By focusing on these dimensions together, the study addresses a gap in the literature, where research on how psychosocial and organizational factors intersect with digital preparedness remains scarce. Given the urgency of supporting an effective and sustainable digital transition in healthcare, investigating these dynamics represents a pressing priority for future studies and for the design of organizational solutions.

This study is particularly important because, while extensive research has examined burnout, bureaucratic overload, and impostor syndrome in healthcare, and several systematic reviews have already mapped barriers and facilitators to digital health adoption among professionals, these efforts have predominantly emphasized technical, infrastructural, and training-related challenges [23]. By contrast, far fewer studies have addressed how socio-demographic, psychological, and organizational factors shape digital preparedness. Our work aims to contribute to this gap by offering new insights into professionals’ experiences with digital transition.

## 2. Materials and Methods

### 2.1. Participants and Procedures

The sample was recruited from healthcare personnel at the Villa Chiara and Villa Erbosa Clinics, both located in Bologna, Italy, and part of the San Donato Group (Italy’s leading private hospital network). Recruitment took place between February and April 2024 and was facilitated through dedicated informational meetings presenting the project to staff, as well as the engagement of nursing coordinators to promote participation. All personnel aged 18 years or older and employed as healthcare workers at either of the two clinics were eligible to participate (N = 860). No additional exclusion criteria were applied. Participation was entirely voluntary, and no random selection procedures were implemented. A total of 111 individuals consented to take part in the study. Of these, 81 (73%) were female and 30 (27%) were male. The group included 12 (11%) physicians, 51 (46%) nurses, 26 (23%) other healthcare workers (e.g., nursing assistants, physiotherapists, laboratory technicians), and 22 (20%) administrative staff members, who were included as part of the healthcare workforce given their central role in managing digital platforms and supporting the functioning of care delivery. Participants completed an online battery of questionnaires, administered via the Qualtrics platform, assessing bureaucratic and administrative workload, the impact of digital transition, and the use of telemedicine and electronic health records. Furthermore, specific assessments were conducted to evaluate burnout, impostor syndrome, and workplace organization.

The study was conducted according to the guidelines of the Declaration of Helsinki and approved by the Ethics Committee (protocol n. RM-2023-757).

### 2.2. Measures

A custom-made socio-demographic questionnaire was used to collect socio-demographic information such as education level, marital status, presence of children, occupation, and annual salary. Additionally, a specific form was created to assess the current working conditions, focusing on bureaucratic and organizational aspects, including the time dedicated to these tasks, the perceived support in managing them, and the potential introduction of new support roles. The questionnaire also aimed to gather information regarding the impact of digital transition on healthcare professionals, evaluating how important this transition is perceived to be, as well as the preparation and skills perceived by the operators, along with the opportunities and obstacles they identify. Moreover, the use of telemedicine and electronic health records was assessed, including how frequently these technologies are utilized, the perceived competence regarding their use, and any potential barriers to their adoption. In addition, psychological assessments were administered, including the Maslach Burnout Inventory (MBI), the Work Organisation Assessment Questionnaire (WOAQ), and the Clance Impostor Phenomenon Scale (CIPS).

The MBI [24,25] consists of 22 items divided into three subscales that assess the three components of burnout: emotional exhaustion (e.g., “I feel emotionally drained from my work”; 9 items), depersonalization (e.g., “I feel tired when I get up in the morning and have to face another day at work”; 5 items), and personal accomplishment (e.g., “I accomplish many worthwhile things in this job”; 8 items). Each item is rated on a seven-point Likert scale (0 = “never”; 6 = “every day”). In this study, we adopted the Italian validated version of the MBI [25], in which the subscales showed good internal consistency (emotional exhaustion: α = 0.87; depersonalization: α = 0.68; personal accomplishment: α = 0.76). The cut-off values used for Italian healthcare workers were those reported in the validation study: emotional exhaustion low (≤14), medium (15–23), high (≥24); depersonalization low (≤3), medium (4–8), high (≥9); personal accomplishment low (≥37), medium (30–36), high (≤29) [25].

The WOAQ [26,27] is a questionnaire comprising 28 items on a five-point Likert scale (5 = very good, 1 = major problem). It is composed of five subscales assessing: quality of relationships with management (items 1–9); reward and recognition (items 10–16); workload issues (items 17–20); quality of relationships with colleagues (items 21–22); and quality of the physical environment (items 23–28). Items assess aspects such as whether staff feel adequately recognized for their work or supported by management in completing tasks. Scoring involves summing the item scores, resulting in a total score ranging from a minimum of 28 to a maximum of 140, with higher scores indicating better quality of work organization. The Italian version of the WOAQ has demonstrated good internal consistency in previous research (α = 0.95) [27].

The CIPS [28,29] is a 20-item self-report scale designed to measure impostor syndrome, which is characterized by feelings of personal incompetence despite external success. Items are rated on a five-point Likert scale (1 = “not at all true” to 5 = “completely true”) and assess cognitions and behaviors related to impostor feelings (e.g., “I often worry that I will be discovered as not being as capable as people think I am”). The total score is calculated by summing responses, yielding a range from 20 to 100, with higher scores indicating more frequent and severe impostor syndrome (≤40 = low; 41–60 = moderate; 61–80 = frequent; ≥81 = intense). The CIPS has demonstrated a high level of internal consistency, with α values ranging from 0.84 to 0.96 [29]. For the present study, the CIPS was translated into Italian using standard forward–backward translation procedures to ensure linguistic and conceptual equivalence.

### 2.3. Statistical Analysis

Summary statistics were computed to describe both qualitative and quantitative variables. Categorical variables are reported as frequencies and percentages, while measures of central tendency and variability were used for quantitative variables and psychological scales.

Classification and Regression Trees (CART) have been applied to identify the variables that best distinguish professionals with moderate vs. high levels of perceived preparedness for the digital transition. Actually CART is a data-driven, nonparametric approach that uses a binary recursive partitioning algorithm and automatically selects relevant variables and optimal cut-off points to differentiate participants based on their level of digital readiness.

At each node, the CART algorithm (i) examines all the variables and (ii) for each variable it finds the best split, then it compares the variable splits and selects the best of these. These two steps are iteratively executed and applied also to each of the daughter nodes until leaf node is obtained and tree growing process is concluded. In choosing the best split, at each node, the criterion of maximizing decrease in impurity is applied. In the analyses we used a standard criterion, i.e., the Gini rule, which is a measure of how well the splitting rule separates the classes contained in the parent node. The underlying idea is that splits are chosen such that obtained sub-groups are internally most homogeneous and externally most heterogeneous. Since tree-based procedures are prone to overfitting, we introduced a constraint during the tree-building phase by fixing the minimum number of observations required in each terminal node at 10. CART is an appealing procedure allowing the derivation of classification rules, which may be helpful for implementing personalized training interventions.

## 3. Results

An overview of participants’ socio-demographic and occupational characteristics is provided in Table 1 while details by professional group are reported in the Supplementary Materials.

### 3.1. Digital Transition

**Figure 1:**
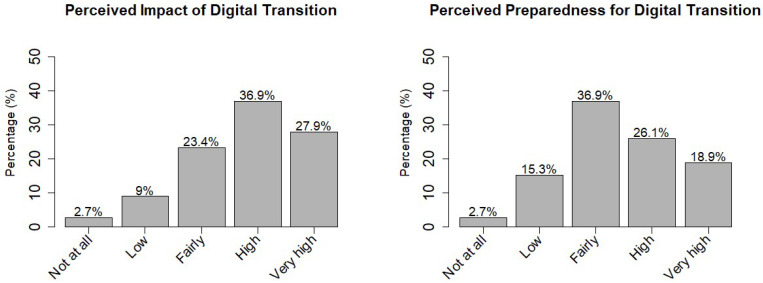
Distribution of responses regarding the perceived impact of the digital transition on professional activity and the perceived preparedness for addressing the digital transition.

As shown in Figure 1, regarding the digital transition, 88% (n = 98) of the sample reported that it impacts their professional activities. When analyzed by professional category, this perception was reported by 91% (n = 20) of administrative staff, 90% (n = 46) of nurses, 85% (n = 22) of other healthcare workers, and 83% (n = 10) of physicians. However, 18% (n = 20) of the sample felt unprepared for the digital transition in their profession, particularly 23% (n = 18) of females compared to 6.7% (n = 2) of males.

Analyzing the data by professional category, administrative staff were the least prepared, with 31.5% (n = 7) reporting feeling unprepared for the digital transition. When exploring the skills needed to facilitate the digital transition, the effective use of platforms and digital tools for patient management was identified as the most important skill to acquire (61%; females 54%; males 80%). Additionally, 61% of the sample (females 63%; males 57%) considered support for technical problem-solving to be the most valuable resource for successfully navigating the digital transition, followed by specific training in digital competencies (60%; females 64%; males 50%).

In exploring the risks associated with the digital transition in healthcare professions, 52% of participants (54% females; 47% males) identified the decreased human interaction and diminished doctor–patient relationship as the primary concern. This apprehension was notably higher among nurses (59%) and physicians (67%). Furthermore, 58% of physicians expressed that excessive reliance on technology represents another potential risk. Seventy-one percent of the sample (70% females; 73% males) believe that the primary opportunity presented by the digital transition is the ability to streamline and automate bureaucratic tasks, thereby allowing for greater focus on patient care.

### 3.2. Bureaucratic and Organizational Aspects

Regarding bureaucratic and organizational aspects, 68% (n = 75) of participants reported spending more time on administrative tasks than on patient care. Additionally, 57% (n = 63) indicated that they do not receive any support for managing bureaucratic tasks, and among these, 73% (n = 46) considered the presence of administrative support as potentially useful. Even among those who already receive assistance, 71% expressed the need for an additional support figure.

### 3.3. Telemedicine

Regarding telemedicine, 34% (n = 28) of females and 23.7% (n = 7) of males of overall sample reported feeling either not at all or only slightly competent in utilizing this technology. Among professions, the analysis reveals that 41% (n = 5) of physicians and 27.8% (n = 14) of nurses also feel either not at all or only slightly competent in its use. Furthermore, 75% (n = 9) of physicians reported that they never conduct telemedicine consultations. In contrast, informal communication methods such as WhatsApp, email, and phone calls are frequently utilized by 75% (n = 9) of the physicians. The primary challenges encountered in using telemedicine involve connection instability and difficulties related to the platform. Additionally, 10% of the sample expressed concerns about the lack of physical contact and the difficulty in establishing a doctor–patient relationship. Conversely, the main factor facilitating the use of telemedicine is organizational flexibility.

### 3.4. Mental Health and Work Organization Quality

Descriptive statistics for the Maslach Burnout Inventory (MBI), the Work Organizational Assessment Questionnaire (WOAQ), and the Clance Impostor Phenomenon Scale (CIPS) are reported in Table 2.

The results of the analysis using the MBI provide an overview of the levels of burnout within the sample. Analysis revealed that 51% of the sample experienced high levels of emotional exhaustion, with a prevalence of 62% (n = 50) among females and 23% (n = 7) among males. Among professions, 61% (n = 31) of nurses experienced high levels of emotional exhaustion, followed by administrative (50%; n = 11), other healthcare workers (50%; n = 13), and physicians (17%; n = 2). Additionally, 53% of the sample reported low levels of personal accomplishment, with 63% of males (n = 19) and approximately 49% (n = 40) of females afflicted. Among professions, 75% (n = 9) of physicians reported low personal accomplishment, followed by nurses with 63% (n = 32), the other healthcare (46%; n = 12) and administration (27%; n = 6) categories. Regarding depersonalization, 41% of the sample exhibited high levels of this feature, with 46% (n = 37) of females and 30% (n = 9) of males reporting such experiences. Moreover, 46% (n = 12) of those classified as other healthcare and 45% (n = 10) of administrative staff showed high levels of depersonalization, followed by 43% of nurses (n = 22) and 17% of physicians (n = 2). Additional details are available in Table 2.

The Clance Impostor Phenomenon Scale (CIPS) showed a mean score of 39.99 (SD = 15.11), with a median of 36.5 (IQR = 28.0–47.0) and observed values ranging from 20 to 100 (Table 2). In terms of clinical categorization, the majority of participants (57%) reported only few impostor experiences, whereas 34% reported moderate, 8% frequent, and 2% intense experiences. When grouping moderate, frequent, and intense categories, approximately 43% of the sample (n = 45) reported impostor syndrome, with a prevalence of 45% (n = 35) among females and 37% (n = 10) among males. When analyzing the data by profession, nurses descriptively reported the highest percentage of impostor syndrome at 56% (n = 26).

The results of the WOAQ indicate no issues related to workload or the physical environment. There appears to be a good relationship with colleagues, as well as with management, and a high level of perceived recognition. When descriptively analyzing the data by professional category, physicians reported higher scores across the various subscales (98.25), while administrative staff reported lower scores (75.36).

### 3.5. Predictors of Perceived Preparedness for Addressing the Digital Transition

Preparedness for the digital transition was further analyzed using a CART algorithm to identify its main predictors. The CART algorithm selected age, MBI emotional exhaustion, and Impostor Syndrome as the best discriminating variables to distinguish between those who feel well-prepared to tackle the digital transition and those who do not.

**Figure 2:**
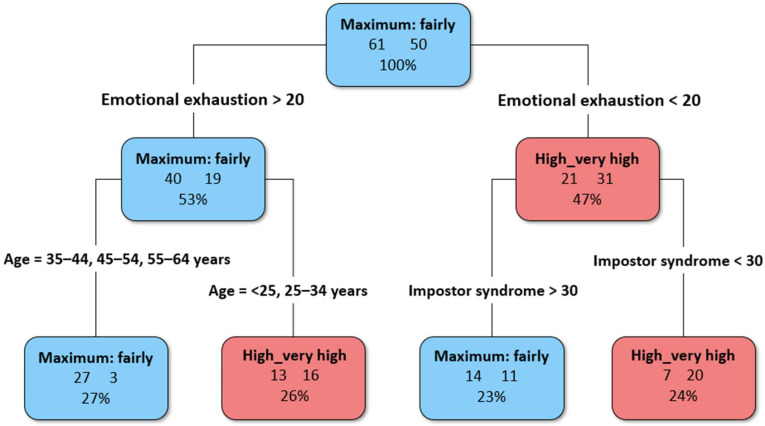
Decision tree for the classification of professionals who feel well-prepared versus not well-prepared to tackle the digital transition.

Emotional exhaustion emerged as the primary splitting variable (Figure 2), thus representing the first psychological construct to evaluate in the classification process. Individuals reporting levels of emotional exhaustion lower than 20 and levels of Impostor Syndrome below 30 were more likely to feel prepared to face the digital transition. Conversely, individuals reporting higher levels of emotional exhaustion (>20) and older than 35 years feeling were the least prepared. Although all the three burnout components, the overall organizational quality of work were included in the model, along with gender and professional information, these variables were not chosen by the procedure, indicating lower discriminative power in this classification.

## 4. Discussion

This research investigated healthcare professionals’ psychological well-being and their preparedness for the digital transition within two San Donato Group facilities, Villa Chiara and Villa Erbosa, both located in the city of Bologna, Italy. The findings offer a nuanced picture of how psychological and organizational factors intersect in shaping healthcare workers’ experiences, with particular attention to bureaucratic workload, digital competence, burnout, and impostor syndrome.

In line with the centrality of digital preparedness highlighted in the introduction, the majority of participants (88%) reported that digital transition already has a tangible impact on their professional activities. This perception was consistent across roles, with particularly high recognition among administrative staff (91%) and nurses (90%). These findings suggest that healthcare professionals are acutely aware of the transformative effect of digital technologies on their daily practice, confirming the relevance of situating digital preparedness as a key dimension of professional well-being.

Despite this, almost one in five participants (18%) reported feeling unprepared for the digital transition, with marked differences by gender and profession. Women (23%) more frequently reported feeling unprepared than men (6.7%). This finding aligns with Sánchez-Canut et al. (2023) [30], whose systematic review shows that women’s professional digital competences are both under-researched and unevenly developed compared to men’s. The authors emphasize that, despite women often demonstrating strengths in areas such as information evaluation and online networking, they remain at higher risk of exclusion in the digital transition. They also stress the lack of sex-disaggregated data in much of the existing research, which limits a nuanced understanding of gender-specific barriers to digital competence. Taken together, these insights suggest that gendered patterns of preparedness cannot be explained merely by sample composition, which in our case was composed predominantly of women. This distribution reflects the demographic reality of the healthcare workforce, where women—especially nurses—represent the majority [31].

Professional differences further reinforce this uneven landscape. Administrative staff felt the least prepared (31.5%), diverging from some prior evidence that often points to physicians and nurses showing lower digital competence compared to other healthcare professions, primarily due to gaps in digital and telemedicine skills [32,33,34]. This discrepancy may reflect differences in training exposure or in the specific digital tools available across professional categories, underscoring the importance of tailoring training programs to the role-specific needs of each category.

When exploring the resources needed to navigate digitalization, participants emphasized practical and organizational aspects. Effective use of platforms for patient management (61%), technical problem-solving support (61%), and targeted digital training (60%) were rated as the most valuable. These findings suggest that professionals view preparedness not only as a matter of individual skills but also as dependent on organizational investment and ongoing support. This is consistent with high-quality evidence from recent systematic reviews, which identify training opportunities and perceived usefulness of technology as central facilitators of digital health adoption [23].

At the same time, participants highlighted significant risks. More than half of the sample (52%) expressed concerns about the erosion of human interaction and the weakening of the doctor–patient relationship, with physicians (67%) and nurses (59%) particularly worried. This finding is in line with the recent literature warning that digital health may transform the patient–physician relationship in undesirable ways: the replacement of face-to-face contact is considered dangerous because it risks missing crucial clinical information when doctors do not “see” the patient, and it may also impose a “burden of invisible work” on patients, who are required to invest additional time and effort in the diagnostic process [35]. Furthermore, 58% of physicians cautioned against excessive reliance on technology. This concern may be linked to the fear that digital tools could add to an already demanding workload. Consistent with prior research, our results confirm that bureaucratic demands absorb a substantial share of healthcare professionals’ time, often at the expense of direct patient care. Excessive administrative workload has long been associated with increased stress, reduced job satisfaction, and lower quality of care [10,18]. Within this context, it is understandable that professionals view digitalization with ambivalence: while it promises efficiency gains, it also raises the possibility of additional tasks and responsibilities, in line with the literature [23].

Telemedicine provides a concrete example of this ambivalence. Despite its potential advantages in terms of organizational flexibility, resource optimization, and remote care delivery [36], only 10% of physicians in our sample reported using telemedicine regularly. Barriers included technical difficulties (e.g., connection instability, platform usability) and relational concerns—like loss of physical contact, challenges to the doctor–patient relationship [37,38]. This confirms the literature showing that adoption of new care modalities is frequently accompanied by role tensions and requires significant changes in professional identity [39].

Psychological factors also play a central role. Our data confirm high levels of burnout, particularly among women and nurses, consistent with evidence of their greater vulnerability in high-pressure healthcare contexts [11,40,41,42,43,44]. As Barello and colleagues [41] found during the pandemic, female gender is significantly associated with higher emotional exhaustion, partly explained by sociocultural pressures such as gender norms and work–family balance challenges [42]. Nurses, similarly, reported higher emotional exhaustion and reduced personal accomplishment, aligning with the literature that identifies this group as especially vulnerable [43]. These findings reinforce the idea that heavy workloads and challenging contexts amplify risks of burnout [44,45]. Importantly, preparedness for digital transition appears to be associated with burnout levels. Participants who reported lower emotional exhaustion levels also reported higher preparedness.

In our sample, approximately 43% of participants reported moderate to frequent experiences of impostor syndrome. This condition thus emerged as a relevant correlate of burnout. Consistent with previous findings [16,46,47], impostor tendencies were associated with greater emotional exhaustion, depersonalization, and reduced personal accomplishment. Women and nurses reported the highest prevalence, confirming prior evidence of their greater susceptibility [46]. According to the existing literature, impostor syndrome is associated with feelings of inadequacy, reduced professional confidence, and difficulties in adapting to change [16,46,47], which may in turn hinder openness to digital transition, underscoring the need for targeted interventions to enhance resilience and skills among healthcare professionals, particularly regarding new technologies [48,49]. In light of this, those with lower levels of impostor syndrome reported feeling more prepared for digital transition.

The CART analysis further clarified these dynamics by identifying emotional exhaustion as the primary predictor of perceived digital preparedness. Among those with high levels of exhaustion, age became a key factor, with younger professionals (< 35 years) more likely to feel ready for digital transition compared to their older counterparts. This aligns with the idea that younger users are often more responsive to digital tools, although recent studies suggest more nuanced patterns of adoption across generations [50]. Conversely, among those with low emotional exhaustion, impostor syndrome played a decisive role: professionals with fewer impostor experiences reported greater preparedness, while those with higher scores felt less equipped. Notably, professional role was not selected by the model as a relevant predictor, suggesting that when psychological variables are taken into account, differences between categories lose explanatory power. This highlights how readiness for digital transition is shaped less by formal role and more by the interplay between exhaustion, self-perceptions of competence, and age-related differences.

The assessment of organizational conditions in our sample highlighted several strengths, including positive relationships with colleagues and management, a high degree of perceived recognition, and no major issues related to workload or the physical environment. These dimensions, which capture the perceived quality of relational, managerial, and environmental support at work, are generally considered protective factors for professionals’ well-being. However, in our analysis, they were not selected by the CART model as predictors of digital preparedness. A likely explanation is that, given the overall positive evaluation in our sample, these aspects did not vary sufficiently to discriminate between more and less prepared professionals. This does not mean that organizational resources are irrelevant; rather, it underscores how, in contexts where the organizational environment is perceived as satisfactory, other psychosocial vulnerabilities—such as emotional exhaustion and impostor syndrome—become more decisive in shaping readiness for digital transition.

Taken together, these findings contribute to the still limited literature on digital transition in healthcare. While much research has focused on adoption rates and infrastructural challenges, our study integrates structural, psychological, and organizational dimensions, showing how they jointly shape professionals’ digital preparedness. Our results extend previous knowledge by highlighting how psychological aspects, particularly emotional exhaustion, become particularly crucial in the context of digital transition, together with impostor syndrome and age. Future interventions should therefore take these aspects into account to promote tailored digital training and invest in supportive practices, ensuring that digital transformation is sustainable for both healthcare systems and professionals.

## 5. Conclusions

In conclusion, this study highlights how digital transition in healthcare is not only a technical or organizational challenge, but also a deeply psychological one. Emotional exhaustion, impostor syndrome, and age emerged as key factors shaping perceived professionals’ preparedness. Importantly, these dimensions do not act in isolation, but rather in a complex interplay that may render certain groups of professionals more vulnerable than others. These insights point to the need for tailored interventions that combine digital training with psychological support, ensuring that transformation processes strengthen, rather than undermine, healthcare professionals’ well-being.

### Limitations

Some limitations of the present research must be acknowledged. First, only 12 physicians (11% of the total sample) responded to the questionnaire, a result in line with the commonly low response rates in physician surveys [48]. Second, the reliance on self-report questionnaires introduces potential biases, including social desirability and recall bias, which may have influenced the accuracy of participants’ responses [49]. Nonetheless, the use of online questionnaires allowed for wider participation, and the digital format has ensured participants’ privacy, which is essential when collecting sensitive data. Evidence supports the efficacy of online surveys in increasing response rates and maintaining data quality in professional populations [50]. Although the study was conducted within only two healthcare institutions affiliated with the San Donato Group, this setting may also be regarded as a strength, as it enabled a focused yet contextually diverse examination of professionals operating under comparable organizational frameworks. Further investigations involving a broader range of institutions and the integration of objective metrics or multi-source data collection are recommended to enhance the external validity and generalizability of the findings.

## Figures and Tables

**Table 1:** Socio-demographic and occupational characteristics of the participants.

Characteristic	n (%)
**Gender**	
Female	81 (73.0%)
Male	30 (27.0%)
**Marital Status**	
Divorced/Separated	7 (6.3%)
In a Relationship, Cohabiting	21 (18.9%)
In a Relationship, Not Cohabiting	17 (15.3%)
Single	21 (18.9%)
Married	44 (39.6%)
Widowed	1 (0.9%)
**Income**	
<20,000 euros annually	28 (25.2%)
>40,000 euros annually	18 (16.2%)
Between 20,000 and 39,999 euros annually	65 (58.6%)
**Children**	
No	56 (50.5%)
Yes	55 (49.5%)
**Education level**	
Middle School Diploma	3 (2.7%)
High School Diploma	31 (27.9%)
Bachelor’s Degree	50 (45.0%)
Master’s Degree/Single Cycle Degree	13 (11.7%)
Doctorate/Specialization	14 (12.6%)
**Profession**	
Other Healthcare	26 (23.4%)
Administrative	22 (19.8%)
Nurse	51 (45.9%)
Physician	12 (10.8%)

**Table 2:** Descriptive statistics for the Maslach Burnout Inventory (MBI), the Work Organizational Assessment Questionnaire (WOAQ), and the Clance Impostor Phenomenon Scale (CIPS). The “Distribution (%)” column indicates the percentage of participants in each category: for MBI, Low—Medium—High; for CIPS, Low—Moderate—Frequent—Intense.

	n	Mean (SD)	Median (IQR)	Range	Distribution (%)
**MBI**					
Emotional Exhaustion	111	23.57 (15.11)	23.00 (11.00, 36.00)	(0, 54)	31–18–51
Personal Accomplishment	111	36.94 (9.99)	39.00 (33.00, 45.00)	(0, 48)	53–25–22
Depersonalization	111	5.82 (6.70)	3.00 (0.00, 10.00)	(0, 30)	45–14–41
**WOAQ**					
Quality of rel. with management	110	27.28 (9.72)	26.50 (20.00, 35.00)	(9, 45)	
Reward and recognition	110	21.96 (6.72)	22.00 (18.00, 26.75)	(7, 35)	
Workload issues	110	10.74 (4.20)	11.00 (7.00, 13.75)	(4, 20)	
Quality of rel. with colleagues	110	7.91 (2.08)	8.00 (7.00, 10.00)	(2, 10)	
Quality of the physical environment	110	16.29 (5.36)	17.00 (12.00, 19.75)	(6, 30)	
WOAQ Total	110	84.18 (24.32)	83.50 (67.50, 100.50)	(28, 136)	
**CIPS**					
IP tot	104	39.99 (15.11)	36.50 (28.00, 47.00)	(20, 100)	57–34–8–2

## Data Availability

In accordance with Ethics Committee approval and participants’ informed consent, data cannot be shared at the individual level. Only aggregated results, as reported in the manuscript, are available.

## Supplementary Materials

The following supporting information can be downloaded at https://www.mdpi.com/article/10.3390/healthcare13202556/s1; Table S1. Gender, age, marital status, occupation, income level analyzed by profession. Table S2. MBI—Burnout Levels Analyzed by Profession; Table S3. Impostor Syndrome Levels Analyzed by Profession; Table S4. Assessment of Work Organization Levels by Profession.
