# Supplementary material for: Preparedness for the Digital Transition in Healthcare: Insights from an Italian Sample of Professionals
Source: Healthcare (Basel). 2025 Oct 10;13(20):2556. doi: 10.3390/healthcare13202556 (PMC12563548; doi:10.3390/healthcare13202556)
Supplement: Supplementary file 1 [file healthcare-13-02556-s001.zip › healthcare-3831330-supplementary.pdf]

Supplementary materials

Table S1. Gender, age, marital status, occupation, income level analyzed by Profession.

Characteristic	Other Healthcare<br>N = 26	Administrative<br>N = 22	Nurse<br>N = 51	Doctor<br>N = 12
Gender				
Female	16 / 26 (62%)	20 / 22 (91%)	42 / 51 (82%)	3 / 12 (25%)
Male	10 / 26 (38%)	2 / 22 (9.1%)	9 / 51 (18%)	9 / 12 (75%)
Marital Status				
Divorced/Separated	2 / 26 (7.7%)	1 / 22 (4.5%)	2 / 51 (3.9%)	2 / 12 (17%)
In a Relationship, Cohabiting	7 / 26 (27%)	6 / 22 (27%)	8 / 51 (16%)	0 / 12 (0%)
In a Relationship, Not Cohabiting	4 / 26 (15%)	2 / 22 (9.1%)	10 / 51 (20%)	1 / 12 (8.3%)
Single	6 / 26 (23%)	3 / 22 (14%)	11 / 51 (22%)	1 / 12 (8.3%)
Married	7 / 26 (27%)	10 / 22 (45%)	19 / 51 (37%)	8 / 12 (67%)
Widowed	0 / 26 (0%)	0 / 22 (0%)	1 / 51 (2.0%)	0 / 12 (0%)
Income				
< 20,000 euros annually	12 / 26 (46%)	8 / 22 (36%)	8 / 51 (16%)	0 / 12 (0%)
> 40,000 euros annually	2 / 26 (7.7%)	3 / 22 (14%)	2 / 51 (3.9%)	11 / 12 (92%)
Between 20,000 and 39,999 euros annually	12 / 26 (46%)	11 / 22 (50%)	41 / 51 (80%)	1 / 12 (8.3%)
Children				
No	17 / 26 (65%)	9 / 22 (41%)	27 / 51 (53%)	3 / 12 (25%)
Yes	9 / 26 (35%)	13 / 22 (59%)	24 / 51 (47%)	9 / 12 (75%)
Education Level				
Middle School Diploma	3 / 26 (12%)	0 / 22 (0%)	0 / 51 (0%)	0 / 12 (0%)
High School Diploma	8 / 26 (31%)	14 / 22 (64%)	9 / 51 (18%)	0 / 12 (0%)
Doctorate/Specialization	1 / 26 (3.8%)	1 / 22 (4.5%)	1 / 51 (2.0%)	11 / 12 (92%)
Master's Degree/Single Cycle Degree	6 / 26 (23%)	5 / 22 (23%)	1 / 51 (2.0%)	1 / 12 (8.3%)
Bachelor's Degree	8 / 26 (31%)	2 / 22 (9.1%)	40 / 51 (78%)	0 / 12 (0%)
Profession				
Other Healthcare	26 / 26 (100%)	0 / 22 (0%)	0 / 51 (0%)	0 / 12 (0%)

Administrative	0 / 26 (0%)	22 / 22 (100%)	0 / 51 (0%)	0 / 12 (0%)
Nurse	0 / 26 (0%)	0 / 22 (0%)	51 / 51 (100%)	0 / 12 (0%)
Physician	0 / 26 (0%)	0 / 22 (0%)	0 / 51 (0%)	12 / 12 (100%)

Table S2. MBI - Burnout Levels Analyzed by Profession.

Characteristic	Other healthcare N = 26	Administrative N = 22	Nurse N = 51	Doctor N = 12
Emotional Exhaustion				
Mean (SD)	23.81 (15.70)	25.86 (16.79)	25.12 (13.96)	12.25 (11.76)
Median (IQR)	23.00 (12.75, 35.75)	22.00 (13.25, 37.75)	26.00 (13.00, 37.00)	10.50 (4.50, 14.50)
Range	0.00, 54.00	3.00, 53.00	2.00, 48.00	0.00, 40.00
Personal Accomplishment				
Mean (SD)	36.31 (11.10)	29.36 (12.22)	39.25 (7.19)	42.33 (5.07)
Median (IQR)	36.50 (31.50, 45.00)	30.50 (22.00, 38.75)	40.00 (35.50, 45.00)	42.50 (38.25, 47.25)
Range	0.00, 48.00	8.00, 48.00	18.00, 48.00	35.00, 48.00
Depersonalization				
Mean (SD)	6.65 (8.27)	5.50 (6.05)	6.27 (6.49)	2.67 (4.27)
Median (IQR)	3.50 (0.00, 10.00)	3.00 (0.00, 10.00)	4.00 (1.00, 11.00)	1.00 (0.75, 2.50)
Range	0.00, 30.00	0.00, 19.00	0.00, 20.00	0.00, 15.00
Emotional Exhaustion				
low	7 / 26 (27%)	6 / 22 (27%)	13 / 51 (25%)	8 / 12 (67%)
medium	6 / 26 (23%)	5 / 22 (23%)	7 / 51 (14%)	2 / 12 (17%)

high	13 / 26 (50%)	11 / 22 (50%)	31 / 51 (61%)	2 / 12 (17%)
Personal accomplishment				
low	12 / 26 (46%)	6 / 22 (27%)	32 / 51 (63%)	9 / 12 (75%)
medium	7 / 26 (27%)	5 / 22 (23%)	13 / 51 (25%)	3 / 12 (25%)
high	7 / 26 (27%)	11 / 22 (50%)	6 / 51 (12%)	0 / 12 (0%)
Depersonalization				
low	12 / 26 (46%)	9 / 22 (41%)	20 / 51 (39%)	9 / 12 (75%)
medium	2 / 26 (7.7%)	3 / 22 (14%)	9 / 51 (18%)	1 / 12 (8.3%)
high	12 / 26 (46%)	10 / 22 (45%)	22 / 51 (43%)	2 / 12 (17%)

Table S3. Impostor Syndrome Levels Analyzed by Profession.

Characteristic	Other healthcare N = 26	Administrative N = 22	Nurse N = 51	Doctor N = 12
IP tot				
Mean (SD)	40.67 (17.68)	39.32 (14.53)	41.34 (14.43)	34.09 (13.49)
Median (IQR)	37.50 (30.75, 42.00)	34.00 (30.25, 41.00)	43.00 (27.50, 51.00)	28.00 (25.50, 41.00)
Range	21.00, 100.00	20.00, 81.00	20.00, 70.00	22.00, 59.00
(Missing)	2	0	4	1
IP intensity				
low	15 / 24 (63%)	15 / 22 (68%)	21 / 47 (45%)	8 / 11 (73%)
frequent	1 / 24 (4.2%)	1 / 22 (4.5%)	6 / 47 (13%)	0 / 11 (0%)
intense	1 / 24 (4.2%)	1 / 22 (4.5%)	0 / 47 (0%)	0 / 11 (0%)
moderate	7 / 24 (29%)	5 / 22 (23%)	20 / 47 (43%)	3 / 11 (27%)
(Missing)	2	0	4	1

Table S4. Assessment of Work Organization Levels by Profession.

Characteristic	Other healthcare N = 26	Administrative N = 22	Nurse N = 51	Physicians N = 12
Quality of relationships with management				
Mean (SD)	24.31 (11.20)	24.36 (7.48)	28.80 (9.32)	32.75 (8.72)
Median (IQR)	25.00 (15.25, 32.00)	23.50 (19.25, 29.50)	29.00 (22.25, 35.75)	32.00 (26.75, 39.25)
Range	9.00, 45.00	11.00, 38.00	11.00, 45.00	20.00, 45.00
(Missing)	0	0	1	0
Reward and recognition				
Mean (SD)	21.08 (6.92)	19.41 (5.37)	22.42 (6.64)	26.67 (6.89)
Median (IQR)	21.50 (17.25, 24.00)	19.50 (16.50, 23.00)	22.50 (18.00, 28.00)	28.50 (21.75, 32.25)
Range	7.00, 35.00	7.00, 28.00	8.00, 35.00	15.00, 35.00
(Missing)	0	0	1	0
Workload issues				
Mean (SD)	10.58 (4.28)	9.64 (3.76)	10.76 (3.92)	13.00 (5.43)
Median (IQR)	10.00 (8.00, 13.50)	10.00 (7.00, 11.00)	11.00 (7.00, 12.75)	14.50 (8.25, 17.25)
Range	4.00, 20.00	4.00, 18.00	4.00, 19.00	4.00, 20.00
(Missing)	0	0	1	0
Quality of relationships with colleagues				
Mean (SD)	6.73 (2.59)	7.73 (1.61)	8.22 (1.90)	9.50 (0.52)
Median (IQR)	7.50 (4.25, 8.75)	8.00 (7.00, 9.00)	9.00 (7.00, 10.00)	9.50 (9.00, 10.00)

Range	2.00, 10.00	4.00, 10.00	3.00, 10.00	9.00, 10.00
(Missing)	0	0	1	0
Quality of the physical environment				
Mean (SD)	16.62 (5.69)	14.23 (4.10)	17.02 (5.40)	16.33 (6.17)
Median (IQR)	16.00 (12.25, 19.75)	15.00 (12.00, 17.75)	17.00 (12.25, 20.75)	17.00 (11.50, 20.50)
Range	6.00, 27.00	6.00, 20.00	7.00, 30.00	7.00, 26.00
(Missing)	0	0	1	0
QOAQ tot				
Mean (SD)	79.31 (27.52)	75.36 (17.59)	87.22 (23.44)	98.25 (25.10)
Median (IQR)	78.50 (63.00, 95.00)	77.00 (65.25, 87.50)	87.50 (71.00, 105.00)	96.00 (77.00, 121.25)
Range	28.00, 136.00	33.00, 105.00	34.00, 133.00	58.00, 136.00
(Missing)	0	0	1	0
